# Failure of the Nemo Trial: Bumetanide Is a Promising Agent to Treat Many Brain Disorders but Not Newborn Seizures

**DOI:** 10.3389/fncel.2016.00090

**Published:** 2016-04-14

**Authors:** Yehezkel Ben-Ari, Philippe Damier, Eric Lemonnier

**Affiliations:** ^1^INMED - Institut National de la Santé et de la Recherche Médicale U901, Aix–Marseille UniversityMarseilles, France; ^2^Institut National de la Santé et de la Recherche Médicale, Centre d'Investigation Clinique 0004Nantes, France; ^3^Hôpital Le Cluzeau, CHU LimogesLimoges, France

**Keywords:** GABA, bumetanide, epilepsy, autism, parkinson disease, clinical trials

## Abstract

The diuretic bumetanide failed to treat acute seizures due to hypoxic ischemic encephalopathy (HIE) in newborn babies and was associated with hearing loss (NEMO trial, Pressler et al., 2015). On the other hand, clinical and experimental observations suggest that the diuretic might provide novel therapy for many brain disorders including Autism Spectrum Disorders (ASD), schizophrenia, Rett syndrome, and Parkinson disease. Here, we discuss the differences between the pathophysiology of severe recurrent seizures in the neonates and neurological and psychiatric disorders stressing the uniqueness of severe seizures in newborn in comparison to other disorders.

Whereas in adult (mature) neurons GABA provides most of the inhibitory tone, in immature neurons it depolarizes and often excites due to higher levels of intracellular chloride ((Cl^−^)_I_) (Ben-Ari et al., 1989, 2007; Ben-Ari, 2014). Extensive investigations suggest that the two chloride co-transporters NKCC1 and KCC2 play a central role in this developmental sequence. NKCC1 and KCC2 are respectively important chloride importers and exporters and the activity of the former early on is thought to underlie the depolarizing and often excitatory actions of GABA on immature neurons (reviewed in Ben-Ari, 2014). High (Cl^−^)_I_ levels, depolarizing and excitatory actions of GABA are also observed after a wide range of insults and disorders including seizures, Autism Spectrum Disorders (ASD), chronic pain, spinal chord lesions, Down's syndrome, brain trauma, cerebral edema formation, cerebral artery occlusion, and diabetic ketoacidosis (Ben-Ari et al., 2007; Boulenguez et al., 2010; Pizzarelli and Cherubini, 2011; Ben-Ari, 2014; Deidda et al., 2014; Kaila et al., 2014). Therefore, drugs that restore low (Cl^−^)_I_ levels may provide novel therapies for a wide range of disorders. The diuretic bumetanide is one such drug that specifically blocks the NKCC1 chloride importer thereby reducing (Cl^−^)_I_ levels and restoring GABAergic inhibition in many neuronal types in physiological and pathological conditions. The recent failure of the NEMO trial aimed at treating seizures in newborn babies with bumetanide (Pressler et al., 2015) has casted some doubts on the therapeutic promises of bumetanide and magnified its side effects. Here, we would like to discuss why this view is not tenable; namely the major differences between the pathogenesis of acute neonatal seizures due to HIE and other disorders, in addition to the good tolerability of bumetanide reported over the last four decades.

NEMO (Pressler et al., 2015) was a phase I/II trial to assess the safety and optimal dose of bumetanide for the treatment of acute neonatal seizures not responding to a loading dose of phenobarbital. Newborn babies were treated with the diuretic in adjunct to a second dose of phenobarbital. Bumetanide did not reach the primary endpoint (≥80% reduction in EEG seizure burden without the need for rescue antiepileptic drugs in >50% of the patients). The trial was terminated prematurely because of hearing loss observed in 3 out of 11 surviving babies. The hearing loss can be ascribed to synergetic effects of several factors such as immaturity of hair cells, differences between the roles of NKCC1 and KCC2 in the regulation of (Cl^−^)_I_ in immature and adult cochlear hair cells, and effects of hypoxic injury and toxicity of aminoglycosides that are aggravated by bumetanide (Brummett, 1981; Lu et al., 1999; Zhang et al., 2007; Abbas and Whitfield, 2009; Milenković and Rübsamen, 2011; Wong et al., 2013; Witte et al., 2014). Pressler and colleagues discuss these issues at length stressing the difficulties of evaluating the potential risks/benefit ratio in babies in severe life threatening conditions. Bumetanide has been used including in preterm babies and young children without ototoxicity previously (Flamenbaum and Friedman, 1982; Laughon et al., 2015). Bumetanide is a well-tolerated diuretic used to treat hypertension and cerebral edema at all ages; its adverse effects are almost entirely restricted to its diuretic effect such as hypotension and hypokalemia (Asbury et al., 1972; Narins and Chusid, 1986) which are usually easy to correct. Therefore, the ototoxic effect seen in the NEMO study is specific to this age group with the additional risk factors mentioned above.

Furthermore, the poor antiepileptic efficacy in the NEMO trial cannot necessarily be extrapolated to other types of seizures. Bumetanide blocks seizures in some animal models but not others (Ben-Ari et al., 2007; Kilb et al., 2007; Nardou et al., 2009; Ben-Ari, 2014) suggesting that the efficacy of bumetanide is seizure type dependent. In addition, GABA exerts excitatory actions in some models of migration disorders but not in others (Isaev et al., 2005; Tyzio et al., 2009; Pressler et al., 2015). For instance, in human resected slices after surgical interventions, bumetanide reduced hyperactivity in temporal lobe epilepsy (Cohen et al., 2006; Pallud et al., 2014) but not in Sturge-Weber Syndrome where GABA inhibits neuronal activity (Tyzio et al., 2009). In a case report, bumetanide reduced seizure burden in adult temporal lobe epilepsies (Eftekhari et al., 2013; Pressler et al., 2015). For clinical trials in neonatal seizures like NEMO, the regulatory authorities and ethic committees request that the administration of a new drug (such as bumetanide) is only given once phenobarbital has failed to stop seizures. Staley and co-workers have advocated the use of bumetanide and phenobarbital as the former augmented the efficacy of the latter (Dzhala et al., 2005, 2008; Wong et al., 2013). However, these observations are significantly hampered by the lack of investigations of the dynamic alterations of chloride regulation that occur with recurrent seizures. Indeed, using a triple chamber with the two interconnected intact hippocampi and their connecting commissures in three different chambers, we have determined the alterations of GABAergic polarity after a determined number of seizures. The regulation of (Cl^−^)_I_ levels was found to be highly seizure history dependent. Bumetanide neither prevented the formation by recurrent seizures of an epileptogenic mirror focus nor efficiently reduced their generation (Nardou et al., 2009, 2011; Milenković and Rübsamen, 2011). Recurrent seizures transformed the actions of GABA from inhibition to excitation and phenobarbital blocked initial seizures but aggravated late ones by reinforcing the excitatory actions of GABA (Figure 1; Abbas and Whitfield, 2009; Nardou et al., 2011). This preparation is not readily transposed to human health hence the need of clinical trials. The logistics of clinical trials in a neonatal intensive care setting as well as the population size of the NEMO trial precludes a detailed analysis of the history of seizures prior to bumetanide administration. In addition, the impossibility to use bumetanide as first intention to treat also strongly hampered the conclusions of the trial. All these factors must be included in any evaluation of antiepileptic actions of bumetanide and in particular the need to use it very shortly after the inaugurating seizures. Collectively they suggest that bumetanide might reduce the severity of some seizures but neither apply to all types nor prevent the long term effects of repeated seizures and as such be inefficient as an antiepileptic agent. In contrast to the results of the NEMO trial, clinical and experimental observations suggest that bumetanide might constitute a therapeutic tool to attenuate several seemingly different brain disorders.

**Figure 1:**
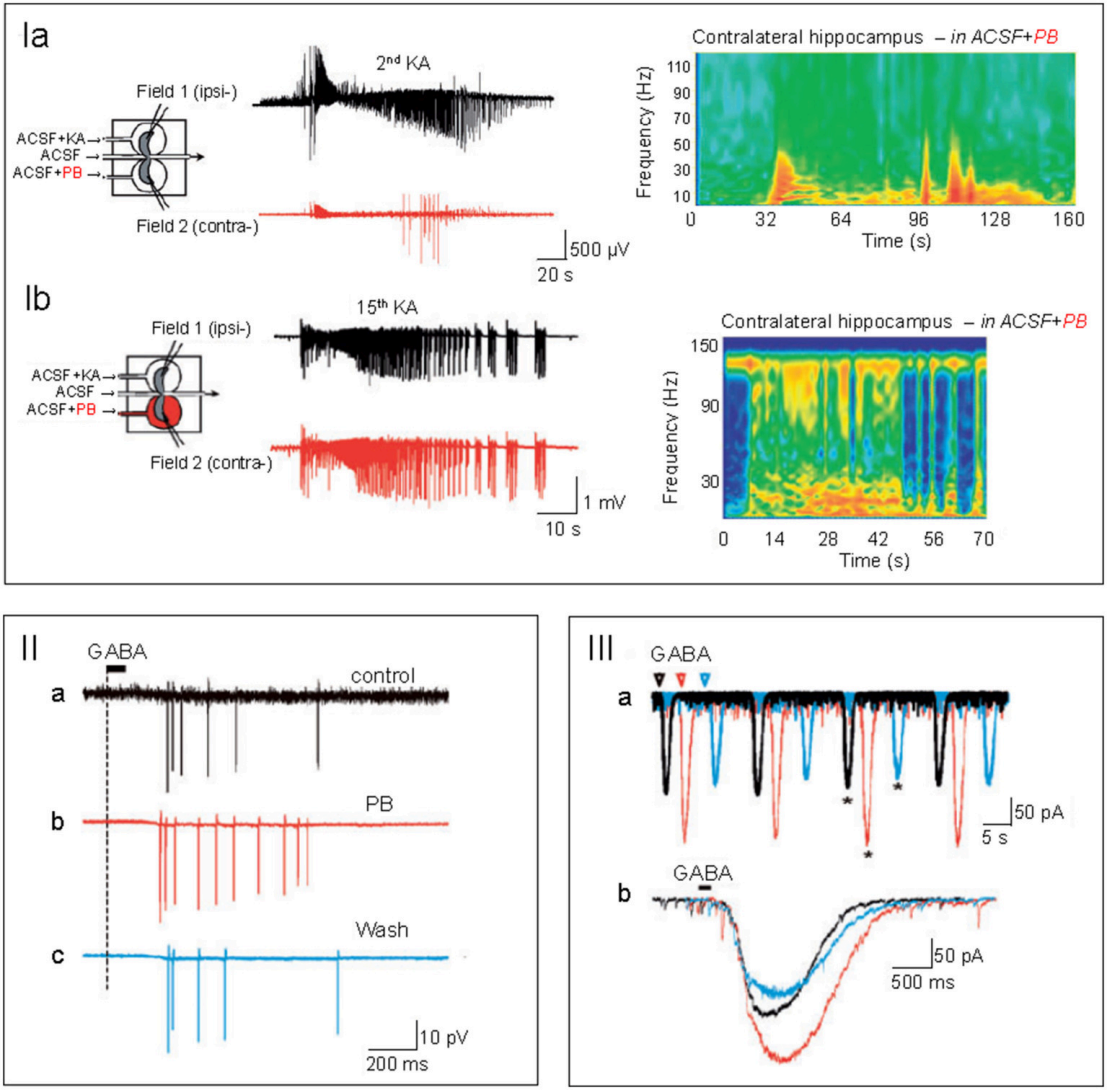
**(I)** Phenobarbital (PB) attenuates early seizures but aggravates late ones. In a triple chamber with the 2 intact hippocampi interconnected by their commissural connections *in vitro*, kainate was applied to one hippocampus generating interictal and ictal discharges in the ipsi-lateral hippocampus (dark trace) that propagated to the contralateral hippocampus.PB application to the contralateral hippocampus (red trace) blocked the initial paroxysmal activity, **(Ia)** but aggravated them when first applied after 15 recurrent ictal and interictal discharges **(Ib)**. Right side: time frequency power plots to illustrate the different effects after a single and any recurrent ictal and interictal discharges. Note the exclusively low frequency events recorded initially in the PB treated hippocampus and the enhanced high frequency events observed after 15 paroxysmal activities. In **(IIa–c)**, spikes evoked in the contralateral hippocampus by focal applications of GABA in the presence of glutamate receptor antagonists. Intact hippocampus subjected to many recurrent IIds and IDs. PB (red) increased the number of spikes in comparison to control (**IIa**-dark) and wash out (**IIc**-blue). **(IIIa,b)** Superimposed perforated-patch recordings of the currents evoked by GABA before (**IIa**-black), during (**IIb**-red), and after PB wash out (**IIc**-blue) in the presence of antagonists of glutamate receptors. Note the enhanced GABA currents produced by PB (taken with permission from Nardou et al., 2011).

## Autism spectrum disorders (ASD)

In naive animals, there is an oxytocin mediated abrupt fall of chloride restricted to the delivery period endowed with neuro-protective actions on the new born brain (Tyzio et al., 2006; Witte et al., 2014). This is abolished in an animal model of ASD and in fragile X (Brummett, 1981; Tyzio et al., 2014) and maternal administration of bumetanide restored in off springs physiological (Cl^−^)_I_ levels and attenuated electrical and behavioral parameters (Eftekhari et al., 2014; Tyzio et al., 2014). Neocortical neurons have elevated (Cl^−^)_I_ levels and excitatory GABA (He et al., 2014). Bumetanide attenuated the severity of ASD in 5–11 years old children in a double blind, randomized study (Lemonnier et al., 2012). Similar observations were made in a pilot case report (Lemonnier et al., 2013). In an open-label trial pilot study in adolescents with ASD treatment with bumetanide was associated with improved emotion recognition and activation of brain regions involved in social and emotional perception in functional magnetic resonance imaging and neuropsychological testing (Hadjikhani et al., 2015). Bumetanide also ameliorated sensory perception in children/adolescents with ASD (Hadjikhani et al., 2015) and altered EEG features (Bruining et al., 2015). Interestingly, in contrast to seizures, benzodiazepines in ASD appear not to be a negative factor for the use of bumetanide; this suggests that chloride-regulating mechanisms are not severely affected in ASD. Behavioral scores of ASD are more efficiently attenuated by a combined educative and bumetanide treatment than by educative/placebo treatment (Du et al., 2015). Interestingly, oxytocin spray that reduces communication deficits (Guastella et al., 2008) act like bumetanide by reducing (Cl^−^)_I_ levels (Tyzio et al., 2006). The effect of bumetanide on seizures that occur in many children with ASD has not been determined as children with epilepsies were not included in these trials.

## Schizophrenia

There is no suitable acceptable animal model of schizophrenia, hence the difficulty in determining chloride equilibrium and intracellular levels. But there is some indirect evidence that chloride co-transporters might be affected. Thus, in human and animal models, there is a reduction of the expression of chloride co-transporters (Arion and Lewis, 2011; Hyde et al., 2011; Kalkman, 2011). Also, drugs known to reduce (Cl^−^)_I_ levels notably oxytocin attenuate schizophrenia symptoms (Cacciotti-Saija et al., 2015; Strauss et al., 2015). In a pilot case study, we tested the effects of bumetanide in a patient suffering from hallucinations and schizophrenia. We found a significant long lasting reduction of the hallucinations; these recur when the treatment was stopped (Lemonnier et al., 2016).

## Parkinson disease

In 2 animal models of Parkinson Disease (PD), striatal medium spiny neurons generated Giant GABAergic Currents that are readily blocked by L–Dopa and lesions of the sub thalamic nucleus suggesting that they constitute relevant signatures of PD (Dehorter et al., 2009, 2012). In a pilot open trial, bumetanide improved gait and reduced freezing in four patients with PD (Damier et al., 2016). Interestingly, bumetanide seems to exert beneficial effects on gait abnormalities and freezing of gait observed in two of these patients (Table 1). These encouraging preliminary observation need to be confirmed in further controlled trials.

**Table 1:** **Effects of bumetanide on Parkinsonian motor symptoms in 4 patients**.

**Cases**	**UPDRS III (OFF)**		**UPDRS II (OFF)**		**Patient's main clinical effect**	**Potassium**		**Side effects**
	Baseline	After 2 mo of bumetanide	Baseline	After 2 mo of bumetanide		Baseline	After 2 mo of bumetanide	
1	44	29	30	18	Improvement of parkinsonism	3.9	3.8	None
2	25	11	15	10	No change	4.1	4.2	Mild polyuria
3^*^	29	26	39	33	Improvement of gait	4.4	4.2	Mild polyuria
								Fatigue
4	43	34	31	26	Improvement of balance	3.8	4.1	Moderate polyuria

*They were treated for 2 months with the diuretic. Reproduced from (Damier et al., 2016)*.

* *Patient 3 was assessed during subthalamic DBS ON. UPDRS indicates Unified Parkinson's Disease Rating Scale; UPDRS III, motor scale in OFF-drug condition; UPDRS II, activities of daily living in the worst conditions (OFF)*.

## Rett syndrome

Rett syndrome includes autistic features and has been often listed in ASD in several studies. Expression of the Mecp2 mutation exclusively in GABAergic interneurons generates several features of Rett syndrome stressing their roles in Rett syndrome pathogenesis (Calfa et al., 2014). In addition, the levels of KCC2 and NKCC1 in the CSF of children with Rett syndrome are respectively reduced and enhanced suggesting an alteration of chloride regulation (Duarte et al., 2013). In human neurons transformed from pluripotent cells of patients with Rett syndrome, there is a deficit in KCC2 and a delay in the excitatory to inhibitory GABA switch (Tang et al., 2016). Furthermore, restoring KCC2 in mecp2 deficient mice rescued GABA deficits.

## Pain

In a variety of experimental conditions, chloride co-transporters are dys-regulated in motor spasticity, morphine-induced hyperalgesia and chronic pain, and KCC2 activating agents attenuate their severity (Boulenguez et al., 2010; Gagnon et al., 2013). Oxytocin like bumetanide attenuated pain in pups by reducing (Cl^−^)_I_ levels in pain pathways (Mazzuca et al., 2011).

In several instances, encouraging preclinical data fail in clinical trials and the reasons for these failures are not always straightforward. They might also lead to the abandon of the animal model although heterogeneity of the clinical data might also be a factor. Yet, with bumetanide, the situation is of particular interest as the convergence of animal and human data is reinforced by a fundamental conceptual series of observations stressing the impact of a wide range of insults on (Cl^−^)_I_ levels paving the way to a common therapeutic strategy to many disorders. Therefore, neither the severe side effects of bumetanide on the hearing system of newborn with hypoxic ischemic encephalopathy (HIE) nor its lack of efficacy in the NEMO study bear relevance to many other disorders. High (Cl^−^)_I_ levels and excitatory GABA play a fundamental role in the pathogenesis of many disorders, generating aberrant activities that perturb the operation of behaviorally relevant oscillations. Bumetanide and other drugs acting on chloride co-transporters can restore low (Cl^−^)_I_ levels in these disorders but may be contraindicated in newborns because chloride co-transporters are still operative.

## Author contributions

PD and EL contributed to the summary on Parkinson disease and autism respectively and YB performed the analysis of the literature and wrote the paper.

## Funding

Experimental work on autism of YB-A was financed by the Bettencourt-Schuller Foundation but grants rejected by the European Research Council. Clinical trials on autism were supported by the Simons Foundation but grants rejected by the Agence Nationale de Recherche.

### Conflict of interest statement

YB is CEO and share holder of Neurochlore 2 and B&A Therapeutics biotech companies dedicated to the development of treatments to autism and parkinson disease. EL and PD are respectively share holders of Neurochlore and B&A Therapeutics.
